# A comparative study between surgical cut down and percutaneous closure devices in management of large bore arterial access

**DOI:** 10.1186/s42155-023-00395-6

**Published:** 2023-10-30

**Authors:** Mohamed Ahmed Mousa, Sherif Samir El Zahwy, Ahmed Fathy Tamara, Wafed Samir, Mahmoud Ahmed Tantawy

**Affiliations:** 1 Department of Cardiology, Faculty of Medicine, Ain Shams University, Cairo, Egypt; 2 Deaprtment of Cardiology, Faculty of Medicine, Misr University for Science and Technology, 6th of October, Egypt

**Keywords:** Transcatheter aortic valve implantation, Endovascular aneurysm repair, Large bore arterial access, Percutaneous closure device, Proglide™, Surgical cutdown

## Abstract

**Background:**

Compared to conventional open surgery, minimally invasive catheter-based procedures have less post procedural complications. Transcatheter aortic valve implantation (TAVI) and endovascular aneurysm repair (EVAR) require large bore arterial access. Optimal site management of large bore arterial access is pivotal to reduce the hospital-acquired complications associated with large bore arterial access. We wanted to compare surgical cutdown versus percutaneous closure devices in site management of large bore arterial access.

**Methods:**

Participants planned for TAVI or EVAR with large bore arterial access more than 10 French were included, while participants with history of bypass surgery, malignancies, thrombophilia, or sepsis were excluded. A consecutive sample of 100 participants (mean age 74.66 ± 2.65 years, 61% males) was selected, underwent TAVI or EVAR with surgical cutdown (group 1) versus TAVI or EVAR with Proglide™ percutaneous closure device (group 2).

**Results:**

The incidence rate of hematoma was significantly lower in group 2 versus group 1 (*p* = 0.014), the mean procedure time (minutes) and the median hospital stay (days) were significantly higher in group 1 versus group 2 (t(98) =  − 2.631, *p* = 0.01, and U = 2.403, *p* = 0.018, respectively), and the c-reactive protein pre-procedure and the c-reactive protein post-procedure were significantly lower in group 2 versus group 1 (U = -2.969, *p* = 0.003, and U = -2.674, *p* = 0.007, respectively).

**Conclusions:**

Our study showed a lower incidence rate of large bore arterial access complications as hematoma, a shorter procedure time, and a shorter hospital stay with percutaneous closure devices compared to surgical cutdown.

## Introduction

Cardiovascular diseases (CVDs) impose a large global and national public health burden. Worldwide around 523 million cases were estimated to have been diagnosed with CVDs and 18.6 million CVD patients were estimated to have died in 2019 [1]. In Egypt, CVDs rank first as the leading cause of premature death among both men and women. In 2017, 46.2% of the overall mortality in Egypt was attributed to CVDs [2]. Hypertension, hypercholesterolemia, diabetes mellitus, and smoking are major risk factors. Patients with hypertension have a higher lifetime risk of CVDs than those without hypertension (63% vs 46%) [3]. Traditionally, medical treatment has been the intervention of choice for chronic CVDs, while thrombolysis, surgery, or minimally invasive catheter-based procedures requiring large bore arterial access has been the standard of care for acute CVDs. Minimally invasive catheter-based procedures requiring large bore arterial access include transcatheter aortic valve implantation (TAVI) and endovascular aneurysm repair (EVAR). Common femoral artery (CFA) access site is the default access site for minimally invasive catheter-based procedures requiring large bore arterial access [4]. Real-world evidence has shown longer hospital stay, greater number of packed RBCs required for transfusion, higher risk of bleeding, vascular, and hospital-acquired complications, and increased short- and long-term mortality rates with minimally invasive catheter-based procedures requiring large bore access [5]. Large bore arterial access complications include arterial pseudoaneurysm with a prevalence rate of 0.05–6% [6], arteriovenous fistula, arterial dissection with a reported incidence rate of 0.06–0.3% and a combined incidence rate of arterial dissection and arterial occlusion of 0.12–0.42% [6], bleeding complications as hematoma with a prevalence rate of 2–12% and retroperitoneal hemorrhage with an incidence rate of 0.15%–0.5% and a mortality rate of 6.6% [6], access site infection with a reported incidence rate of less than 1% [7], and vascular closure devices related complications. Overall, the incidence rate of bleeding and vascular complications associated with large bore arterial access is 20% in TAVI and 12–22% in EVAR [8]. Optimal site management of large bore arterial access is pivotal to reduce the bleeding, vascular, and hospital-acquired complications associated with large bore arterial access. There are 2 main methods used for site management of large bore arterial access: surgical cutdown and percutaneous closure devices. With the increase in number and complexity of the minimally invasive catheter-based procedures, vascular access closure devices have been developed to replace surgical cutdown, achieve effective hemostasis and time management, and manage access site for effective closure with fewer vascular complications [9]. We wanted to compare surgical cutdown versus percutaneous closure devices in site management of large bore arterial access.

## Methods

### Study design

Our study was a 2-year prospective, multicenter, non-randomized, controlled pilot study conducted at two catheterization laboratories in two tertiary care hospitals. This study was performed in accordance with the Egyptian National Commission for Bioethics (National Commission for UNESCO) statement on ethical conduct in human research, study procedures were carried out following the Code of Ethics of the World Medical Association (Declaration of Helsinki), study design and protocol were reviewed and approved by the human ethics committees of Ain Shams University and Misr University for Science and Technology, study participants signed written informed consents, study data was anonymized, and the privacy rights of the study participants were observed diligently.

### Study participants

Study participants were structural heart and cardiovascular disease patients, candidate for TAVI or EVAR, and referred to the catheterization laboratory. The study participants were subjected to history taking and data collection for age, gender, hypertension, diabetes mellitus, family history of ischemic heart disease, dyslipidemia, smoking, and peripheral vascular disease. In addition, study participants were subjected to comprehensive clinical examination including 2 office systolic and diastolic blood pressure measurements while sitting and relaxed, and separated by 3 min, body weight, height, body mass index, examination for pallor, cyanosis, and/or lymph node enlargement, cardiac examination for cardiomegaly, previous cardiac surgery, and/or abnormal auscultatory findings as murmur, pulmonary rales, and/or pericardial rub, 12-lead electrocardiogram (ECG), lower limb arterial duplex, transthoracic echocardiography (TTE), coronary angiography, complete blood count, coagulation profile, serum creatinine, and C-reactive protein (CRP) [10]. Participants with genetic hemostatic disorders (coagulopathies) were excluded from the study.

### Study procedures

We recruited 108 eligible study participants from two hospitals in one country from October 2021 through April 2023. Four study participants died secondary to COVID-19 and 4 study participants were lost to follow up, so the final number of study participants who were enrolled and participated in this study were 100 study participants. The 100 study participants who enrolled and participated in the study were consecutively assigned with an unequal allocation ratio into an unblinded fashion and divided into 2 groups of 43 participants for surgical cutdown (Group 1) versus 57 age and gender frequency matched participants for Proglide™ percutaneous closure device (Group 2).

There were 6 study participants with anatomical abnormalities. Grading of calcification at the access site for surgical cutdown versus percutaneous closure device was based on the peripheral artery calcification scoring system guided by CT aortography.

The surgical cutdown was performed by the vascular surgeon, the procedure time was the time that starts with cutting a 3-cm vertical incision just above the inguinal crease and ends with closing the edges of the arteriotomy with Purse-String 5–0 Prolene Suture in an interrupted or running fashion, the number of sutures for the surgical cutdown was 4–5, and in case of an earlier operated groin, the operators would prefer surgical cutdown.

The percutaneous closure was performed as follows: The Perclose Proglide™ Suture-Mediated Closure (SMC) System is a percutaneous closure device composed of a plunger, handle, guide, and sheath. It tracks over a guidewire and delivers a single monofilament polypropylene suture for closure of large bore arterial access site. A needle, a foot, and a marker lumen are enclosed within the guide. The marker lumen allows for back bleeding and ensures proper device positioning. The plunger advances the needle enclosed within the guide and retrieves the single monofilament polypropylene suture. A hemostasis valve is housed within the sheath to seal the blood flow. A knot pusher and suture trimmer are included to position the tied suture knot on top of the arterial access site and trim the trailing limbs of suture. Before deployment, the marker lumen is flushed, the Proglide™ percutaneous closure device is inserted over the guide wire then the guide wire is removed when its’ exit port reaches the skin level, and the Proglide™ percutaneous closure device is advanced until pulsatile flow is observed from the marker lumen. Next step is suture deployment. The Proglide™ percutaneous closure device is advanced, and the lever lifted to open the foot. Then the Proglide™ percutaneous closure device is stabilized at 45°, retracted until the foot is apposed securely against the vessel wall, and the plunger is depressed to deploy the needles. Then the plunger is pulled back to deploy suture then the suture is pulled taut and cut. Finally, the lever is lowered to close the foot, the Proglide™ percutaneous closure device is allowed to relax, and the lever is returned to the original position. After suture deployment, the suture is managed as follows: the Proglide™ percutaneous closure device is retracted until the guide wire’s exit port reaches the skin level, the suture limbs are removed, the guide wire is inserted into the guide wire exit port, the Proglide™ percutaneous closure device is removed while pulling tension on the suture’s blue limb, the knot pusher is loaded on the suture’s blue limb while applying constant tension to advance the suture’s knot down, hemostasis is assessed, the guide wire removed while maintaining tension on the suture’s blue limb, then the knot is locked by pulling on the suture’s white limb. Finally, the right thumb pulls back the suture trimmer, the right index pulls back the lever, and the suture is cut. For the percutaneous closure, the procedure time was the time that starts with advancing the Proglide™ percutaneous closure device through the large bore arterial access until pulsatile flow is observed from the marker lumen and ends with cutting the deployed suture, the number of sutures was 1–2, and there were no additional devices needed.

Large bore arterial access vascular complications were assessed as follows: bleeding and superficial hematoma were defined/assessed clinically, deep hematoma was defined/assessed by ultrasound, retroperitoneal hemorrhage and arteriovenous fistula were defined/assessed clinically and by ultrasound, arterial pseudoaneurysm was defined/assessed by duplex ultrasound, femoral artery dissection and/or stenosis were defined/assessed by intra procedural angiography, and femoral artery occlusion was defined/assessed clinically, by duplex ultrasound, and/or by intra procedural or post procedural angiography. Large bore arterial access complications were resolved by pharmacotherapy and resuscitation measures. If pharmacotherapy and resuscitation measures failed, we would resolve them by surgical drainage procedure.

### End points

The primary end points of the study were the periprocedural vascular complications (bleeding, femoral artery dissection, stenosis, occlusion, and hematoma), hemoglobin drop and number of packed red blood cells required, presence of local vascular complications during hospital stay and at three months post-procedure, prolonged hospital stay secondary to vascular complications, time required for vascular access closure, and late complications (arterial PA, groin infection, and transient nerve injury). Secondary end point was the major adverse cardiovascular events (MACE) including ischemic stroke, myocardial infarction, and cardiovascular death during hospital stay and at three months post-procedure.

### Statistical analysis

Our study is a non-randomized controlled pilot study. The minimum anticipated observed effect size (correlation coefficient) couldn’t be estimated as there were no previously published or ongoing national studies that assessed the efficacy of percutaneous closure devices in management of large bore arterial access in structural heart and cardiovascular disease patients undergoing TAVI or EVAR. Accordingly, the minimum number of the study participants to be recruited (sample size) was based on feasibility. The assessment outcomes were coded, and the data was analysed with the Statistical Package for the Social Sciences software (SPSS®) version 20. Shapiro wilks test was used to assess normality of data. Quantitative data was expressed as means, standard deviations, and ranges, while qualitative data was expressed as numbers, frequencies, and percentages. Comparisons between parametrically distributed quantitative variables were done with independent t-test or analysis of variance (ANOVA) test, between non-parametrically distributed quantitative variables with Mann–Whitney test, and between qualitative variables with Chi-square test or Fisher Exact test, respectively [11, 12]. The confidence interval was set to 95% and the margin of error accepted was set to 5%. Any comparison considered statistically significant was at *P* < 0.05 or less and highly significant at *P* < 0.01. Final data analysis was as per intention to treat (ITT) principle.

## Results

### Sociodemographic features and baseline characteristics

The 2 study groups were balanced with regards to the sociodemographic features and baseline characteristics (Table 1). The key sociodemographic feature of the enrolled participants was male predominance (62.8% of group 1 and 59.6% of group 2 were males). Age was not significantly different between both groups (mean age was 74.24 ± 2.22 years for group 1 versus 75.08 ± 2.98 years for group 2, P 0.113). Baseline characteristics as weight, height, body mass index, hypertension, diabetes mellitus, smoking, peripheral vascular disease, and dyslipidemia were equally distributed (frequency matched) among both studied groups. All enrolled participants completed the study and there were no withdrawals.

**Table 1:** Comparison between Surgical Group and Proglide™ Group regarding Sociodemographic features and baseline characteristics

		Surgical Group No. = 43		Proglide™ Group No. = 57		Test value*	*P*-value	Sig.
Age	Mean ± SD	74.24 ± 2.22		75.08 ± 2.98		-1.598•	0.113	NS
	Range	71 – 78		70 – 83				
Sex	Female	16 (37.2%)		23 (40.4%)		0.102	0.750	NS
	Male	27 (62.8%)		34 (59.6%)				
		Surgical Group No. = 43		Proglide™ Group No. = 57		Test value*	*P*-value	Sig.
Weight	Mean ± SD	70.84 ± 11.28		73.18 ± 12.97		-0.962	0.338	NS
	Range	51 – 90		53 – 115				
Height	Mean ± SD	167.20 ± 7.56		167.28 ± 14.34		-0.035	0.972	NS
	Range	154 – 183		81 – 185				
Body Mass Index	Mean ± SD	25.19 ± 3.26		27.66 ± 15.96		-1.074	0.285	NS
	Range	20.31 – 33.67		19.96 – 134.13				
		No.	%	No.	%	Test value*	*P*-value	Sig.
Hypertension	Negative	8	18.6%	10	17.5%	0.019	0.891	NS
	Positive	35	81.4%	47	82.5%			
Diabetes Mellitus	Negative	16	37.2%	25	43.9%	0.448	0.503	NS
	Positive	27	62.8%	32	56.1%			
Smoking	Negative	27	62.8%	33	57.9%	0.245	0.621	NS
	Positive	16	37.2%	24	42.1%			
Peripheral Vascular Disease	Negative	37	86.0%	49	86.0%	0.000	0.991	NS
	Positive	6	14.0%	8	14.0%			
Dyslipidemia	Negative	23	53.5%	36	63.2%	0.947	0.330	NS
	Positive	20	46.5%	21	36.8%			

*P*-value > 0.05: Non-significant(NS); *P*-value < 0.05: Significant (S); *P*-value < 0.01: highly significant (HS) *: Chi-square test, •: Independent t-test

### Difference in baseline hemoglobin, post-procedure hemoglobin, hemoglobin drop and number of packed red blood cells required for transfusion between surgical cutdown versus percutaneous closure devices

**Fig. 1:**
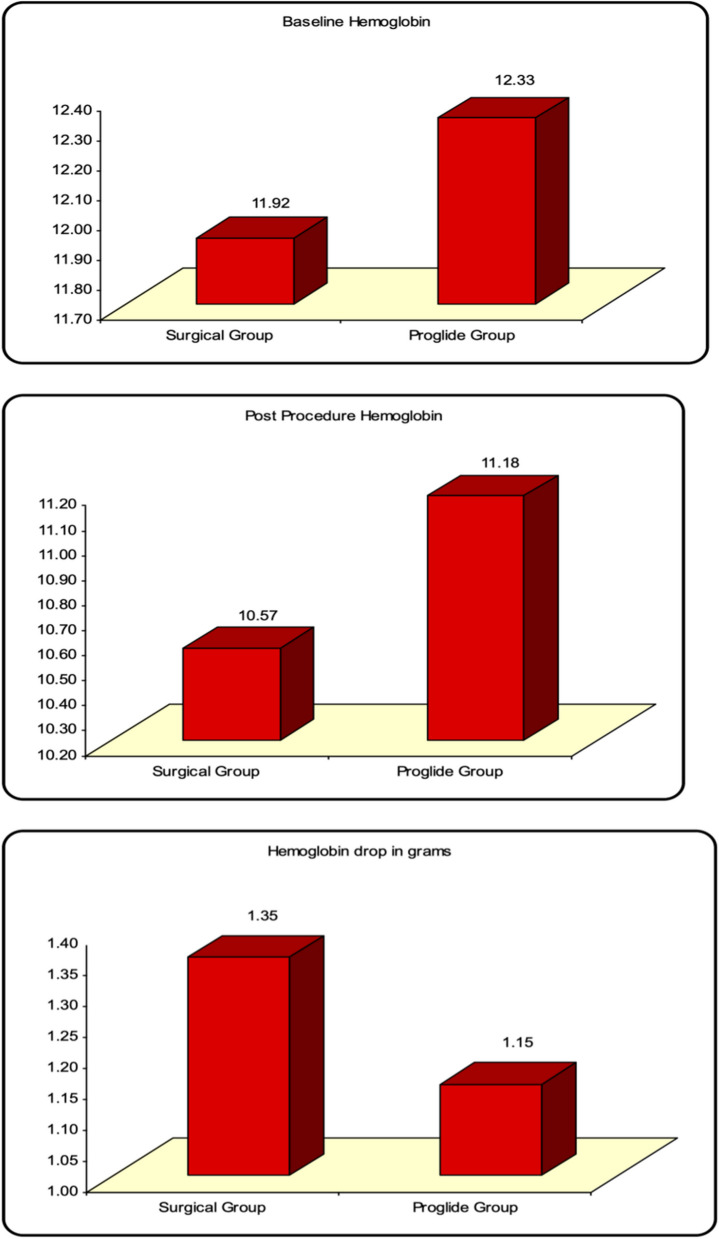
Significant Difference between Surgical Group and ProglideTM Group regarding Baseline Hemoglobin, Post-procedure Hemoglobin, and Hemoglobin Drop

**Fig. 2:**
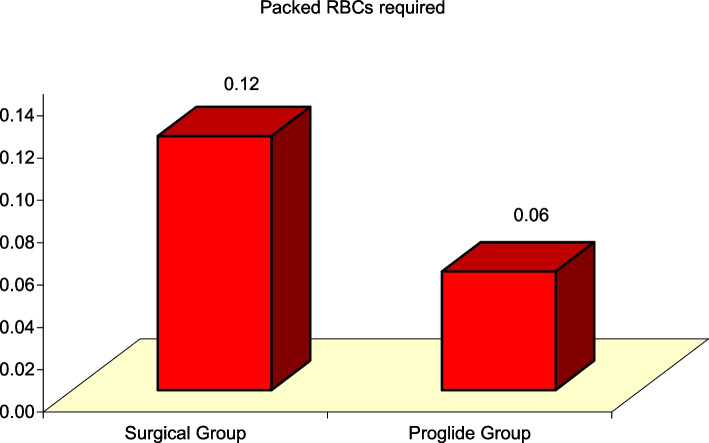
Significant Difference between Surgical Group and ProglideTM Group regarding number of Packed RBCs required for transfusion

Baseline hemoglobin and post-procedure hemoglobin were significantly lower in the Surgical Cutdown group vs Proglide™ group (11.92 ± 1.00 gm/dl vs 12.33 ± 1.06 gm/dl, t(98) = -1.948, *P* = 0.044 and 10.57 ± 1.26 gm/dl vs 11.18 ± 1.04 gm/dl, t(98) = -2.631, *P* = 0.01, respectively), while hemoglobin drop and number of packed red blood cells required for transfusion were significantly higher in the Surgical Cutdown group vs Proglide™ group (1.35 ± 0.53 gm/dl vs 1.15 ± 0.29 gm/dl, t(98) = 2.403, *P* = 0.018 and 0.12 ± 0.33 vs 0.06 ± 0.99, t(98) = 2.975, *P* = 0.004, respectively) (Figs. 1 and 2).

### Association of hematoma with site management of large bore arterial access

**Fig. 3:**
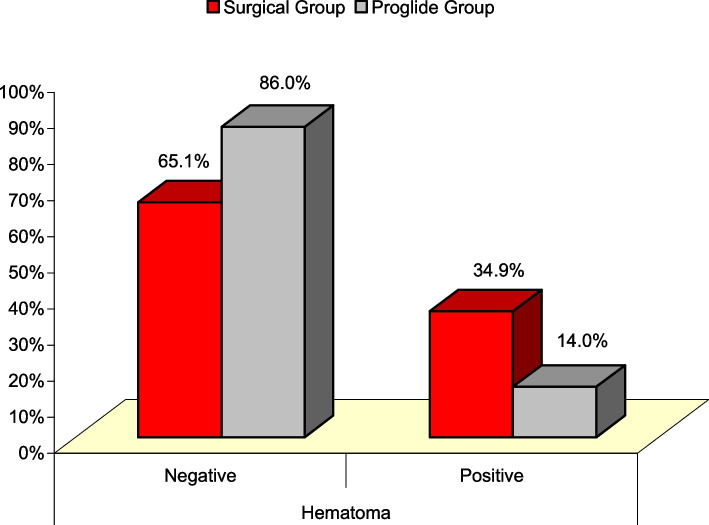
Significant Difference between Surgical Group and ProglideTM Group regarding post-procedure hematoma

Post-procedure hematoma was significantly associated with surgical cutdown in the study population. The incidence rate of post-procedure hematoma was significantly higher in the Surgical Cutdown group compared to the Proglide™ group (34.9% vs 14.0%, Χ^2^ (98) = 6.018, *P* = 0.014) (Fig. 3).

### Difference in procedure time between surgical cutdown versus percutaneous closure devices

**Fig. 4:**
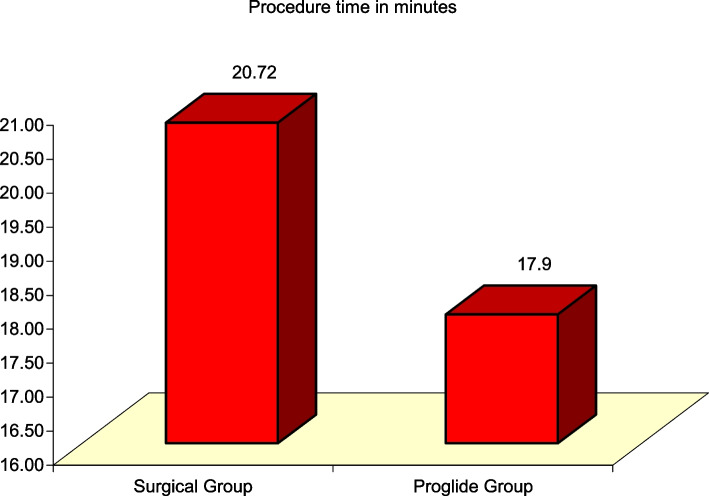
Significant Difference between Surgical Group and ProglideTM Group regarding procedure time in minutes

Procedure time was significantly longer in Surgical Cutdown group vs Proglide™ group (20.72 ± 3.41 min vs 17.90 ± 3.07 min, t(98) = 2.631, *P* = 0.01) (Fig. 4).

### Association of hospital stay with site management of large bore arterial access

**Fig. 5:**
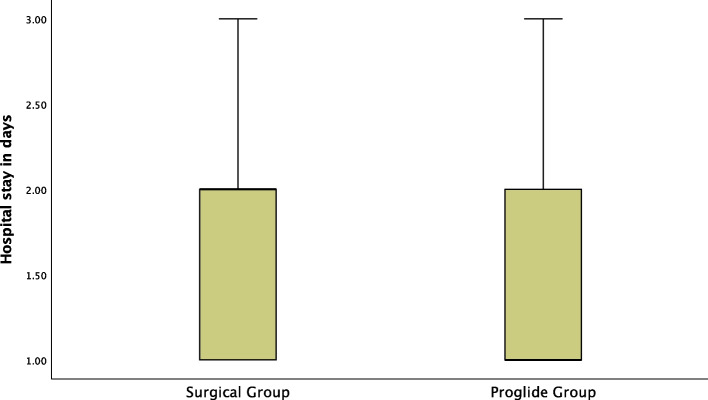
Significant Difference between Surgical Group and ProglideTM Group regarding hospital stay in days

A Mann–Whitney test showed a statistically significant association between hospital stay and surgical cutdown. Hospital stay was significantly longer in the surgical cutdown group (mean rank hospital stay of 2 days for surgical cutdown group vs 1 day for Proglide™ group, U = 2.403, *P* = 0.018) (Fig. 5).

### Association of pre-procedure C-reactive protein and post-procedure C-reactive protein with site management of large bore arterial access

**Fig. 6:**
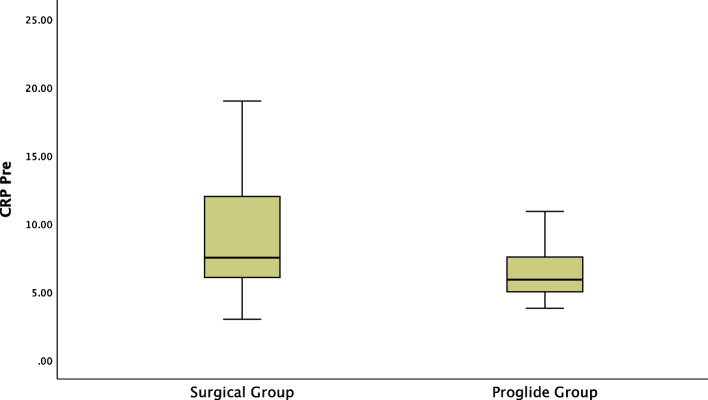
Significant Difference between Surgical Group and ProglideTM Group regarding pre-procedure C-reactive protein

**Fig. 7:**
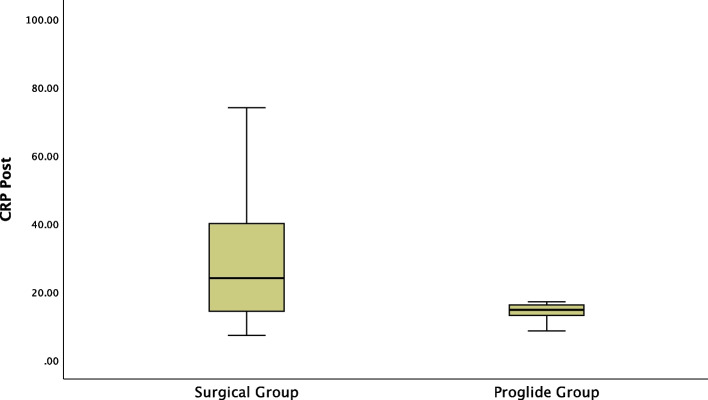
Significant Difference between Surgical Group and ProglideTM Group regarding post-procedure C-reactive protein

A Mann–Whitney test showed significant association between pre-procedure C-reactive protein and post-procedure C-reactive protein with surgical cutdown. Pre-procedure C-reactive protein and post-procedure C-reactive protein were significantly higher in the surgical cutdown group (mean rank pre-procedure C-reactive protein of 7.25 mg/dl for surgical cutdown group vs 5.9 mg/dl for Proglide™ group, U = -2.969, *P* = 0.003, and mean rank post-procedure C-reactive protein of 24 mg/dl for surgical cutdown group vs 14.65 mg/dl for Proglide™ group, U = -2.674, *P* = 0.007, respectively) (Figs. 6 and 7).

## Discussion

Our study reported a significantly longer procedure time in the surgical cutdown group vs Proglide™ group (20.72 ± 3.41 min vs 17.90 ± 3.07 min, t(98) = 2.631, *P* = 0.01).

A recent prospective study by Eckner et al. compared the clinical outcome of 338 patients who underwent TAVI, and the access site was managed with surgical cutdown, versus 449 patients who underwent the same procedure, and the access site was managed with Perclose Proglide™ Suture-Mediated Closure System® (Abbott Vascular). The procedure time was significantly shorter in favour of the percutaneous vascular access closure device (*p* < 0.001) [13]. Another study by Lee et al. reported longer procedure time for EVAR managed with surgical cutdown compared to the same procedure managed with the Perclose Proglide™ vascular access closure device [14]. In 2022, Singh et al. showed significantly lower procedure time in the percutaneous vascular access closure device group (*p* < 0.001). The groups studied were 30 patients who underwent endovascular graft placement using femoral artery approach and access site was managed with a single suture based vascular access closure device (Proglide™) versus 30 patients who underwent the same procedure and access site was managed with surgical cutdown [12]. In addition, the statistically significant finding of shorter procedure time with Proglide™ compared to surgical cutdown (*P* < 0.05) is consistent with the results of Hu et al. and Ichihashi et al. studies [15, 16].

Our prospective study revealed a significant difference in hospital stay between both groups. A Mann–Whitney test showed a statistically significant longer hospital stay in the surgical cutdown group (mean rank hospital stay of 2 days for surgical cutdown group vs 1 day for ProglideTM group, U = 2.403, *P* = 0.018). The statistically significant finding of shorter hospital stay with Proglide™ compared to surgical cutdown (*P* < 0.05) is consistent with the results of Drafts et al., Eckner et al., Ichihashi et al., Lee et al., and Singh et al. studies [12–14, 16, 17].

At baseline, the pre-procedure hemoglobin level and the pre-procedure C-reactive protein were significantly associated with surgical cutdown. The pre-procedure haemoglobin level was significantly lower (11.92 ± 1.00 gm/dl vs 12.33 ± 1.06 gm/dl, t(98) = -1.948, *P* = 0.044) and the pre-procedure C-reactive protein was significantly higher (mean rank pre-procedure C-reactive protein of 7.25 mg/dl for surgical cutdown group vs 5.9 mg/dl for Proglide™ group, *U* = -2.969, *P* = 0.003) in the surgical cutdown group compared to the Proglide™ group, indicating heterogeneity of the study population.

Complications of the surgical cutdown approach, such as post-procedural hematoma or localised cellulitis, are not well documented in the literature, and the actual complication rate for the surgical cutdown approach might be higher. According to Hu et al., (2015), benefits of the Perclose Proglide™ vascular access closure device include improved patient comfort and earlier ambulation resulting in quicker recovery and hospital discharge, less complications associated with prolonged bed rest, and better patient turnover [15]. In 2016, Lee et al. study results showed higher blood loss volumes and higher incidence rate of post-procedural hematoma in the surgical group compared to the Proglide™ group [14]. In 2022, Singh et al. prospective study concluded that the periprocedural complications as infection and hematoma associated with minimally invasive catheter-based procedures were less common with Proglide™ compared to surgical cutdown. They also reported a higher incidence rate of nerve injury in the surgical group [12]. Studies by Al-Khatib et al. and Nelson et al. showed post-procedural CFA complication rate ranging from 8%—22.8%, with an 8% wound infection rate and a 6.5% wound complication rate [18, 19]. In 2016, Ichihashi et al. reported an asymptomatic femoral artery dissection and femoral artery pseudoaneurysms requiring surgical repair overall incidence rate of 1.92% in patients using Proglide™ for EVAR [16]. We reported a significant difference in the incidence rate of post-procedural complications between both groups, with a significantly lower haemoglobin level at baseline and post-procedure in the surgical cutdown group compared to the Proglide™ group (11.92 ± 1.00 gm/dl vs 12.33 ± 1.06 gm/dl, t(98) = -1.948, *P* = 0.044 and 10.57 ± 1.26 gm/dl vs 11.18 ± 1.04 gm/dl, t(98) = -2.631, *P* = 0.01, respectively), as well as significantly higher haemoglobin drop reported in the surgical group versus the Proglide™ group (1.35 ± 0.53 gm/dl vs 1.15 ± 0.29 gm/dl, t(98) = 2.403, *P* = 0.018), respectively. Furthermore, the Proglide™ group required fewer packed RBCs than the surgical group (0.12 ± 0.33 vs 0.06 ± 0.99, t(98) = 2.975, *P* = 0.004), the incidence rate of post-procedure hematoma was significantly higher in the surgical cutdown group compared to the Proglide™ group (34.9% vs 14.0%, Χ^2^ (98) = 6.018, *P* = 0.014), and the post-procedure C-reactive protein was significantly higher in the surgical cutdown group (mean rank post-procedure C-reactive protein of 24 mg/dl for surgical cutdown group vs 14.65 mg/dl for Proglide™ group, U = -2.674, *P* = 0.007).

### Strengths and limitations

Our study was a prospective study that didn’t have missing data allowing robust per protocol analysis and the investigators who analyzed and reported the anonymous data for the end points were blinded to the identity and clinical data of the study participants and hence minimizing observer bias. Instead of using the US guidance for CFA location/puncture site, we used the fluoroscopic guidance for localization of CFA access site [20]. On the other hand, the study has limitations. It was a multicentered non-randomized cohort study with a small sample size (100 study participants). We chose a non-randomized study design as selection of the study participants and the decision for site management of large bore arterial access, whether surgical cutdown or percutaneous Perclose Proglide™ vascular access closure device, was entirely operator dependent and based on the anatomic criteria and comorbidities of the study participants or the complexity of the planned minimally invasive catheter-based procedure, which allowed for selection bias and might have led to favorably selecting the percutaneous Perclose Proglide™ vascular access closure device. The minimum anticipated observed effect size (correlation coefficient) couldn’t be estimated as there were no previously published or ongoing national studies that assessed the efficacy of percutaneous closure devices in management of large bore arterial access in structural heart and cardiovascular disease patients undergoing TAVI or EVAR. Accordingly, the minimum number of the study participants to be recruited (sample size) was small and based on feasibility. Our study showed that the study participants selected for the Proglide™ group had significantly higher hemoglobin and lower pre-procedural C-reactive protein at base line compared to the surgical group. This might end in better post-procedural Proglide™ results. Despite that the minimum number of the study participants to be recruited (sample size) was small, based on feasibility, and the study design was non-randomized which allowed for selection bias that might influence the results of our study, we consider this study a supportive study for systematic reviews and meta-analysis to compare the effectiveness of surgical cut down versus percutaneous closure device in management of large bore arterial access. In addition, being a short study with lack of lengthy follow up didn’t allow us to investigate the chronological relationship between site management of large bore arterial access and the long-term all-cause morbidity (including reintervention or revision surgery) and mortality following structural heart and endovascular interventional procedures.

## Conclusions

Minimally invasive catheter-based procedures require large bore arterial access. Compared to the surgical cutdown approach, TAVI or EVAR using the percutaneous Perclose Proglide™ vascular access closure device achieved effective and safe hemostasis with a significantly lower incidence rate of periprocedural complications such as hematoma or infection, a shorter procedure time, a shorter hospital stay, a lower hemoglobin drop, and a lower need for post-procedure blood transfusion in the study participants. We recommend larger prospective studies with bigger sample size and longer follow-up to assess the chronological relationship between site management of large bore arterial access in TAVI or EVAR and the long-term all-cause morbidity and mortality following Structural Heart and Endovascular Interventional Procedures.

## Data Availability

The data that support the findings of this study are available from the corresponding author upon reasonable request.

## References

[1] Roth GA, Mensah GA, Johnson CO, et al. Global Burden of Cardiovascular Diseases and Risk Factors, 1990–2019: Update From the GBD 2019 Study. J Am Coll Cardiol. 2020;76(25):2982–3021. 10.1016/j.jacc.2020.11.010. 10.1016/j.jacc.2020.11.010 PMC7755038 33309175

[2] Hassanin A, Hassanein M, Bendary A, Maksoud MA. Demographics, clinical characteristics, and outcomes among hospitalized heart failure patients across different regions of Egypt. Egypt Heart J. 2020;72(1):49. Published 2020 Aug 13. 10.1186/s43044-020-00082-0. 10.1186/s43044-020-00082-0 PMC7426340 32789717

[3] Rapsomaniki E, Timmis A, George J (2014). Blood pressure and incidence of twelve cardiovascular diseases: lifetime risks, healthy life-years lost, and age-specific associations in 1·25 million people. Lancet.

[4] Barbetta I, van den Berg JC (2014). Access and hemostasis: femoral and popliteal approaches and closure devices-why, what, when, and how?. Semin Intervent Radiol.

[5] Barbash IM, Barbanti M, Webb J (2015). Comparison of vascular closure devices for access site closure after transfemoral aortic valve implantation. Eur Heart J.

[6] Kolluri R, Fowler B, Nandish S (2013). Vascular access complications: diagnosis and management. Curr Treat Options Cardiovasc Med.

[7] Amato B, Compagna R, De Vivo S, et al. Groin Surgical Site Infection in Vascular Surgery: Systemic Review on Peri-Operative Antibiotic Prophylaxis. Antibiotics (Basel). 2022;11(2):134. Published 2022 Jan 20. doi:10.3390/antibiotics11020134. 10.3390/antibiotics11020134 PMC8868080 35203737

[8] van Wiechen MP, Ligthart JM, Van Mieghem NM (2019). Large-bore Vascular Closure: New Devices and Techniques. Interv Cardiol.

[9] Singh G, Scalise F, Bianchi P (2022). Sheath Size Up and Down With Single Proglide - A Technique for Achieving Hemostasis With Use of Large Size Delivery System During Endovascular Graft Placement. Ann Vasc Surg.

[10] Schlant RC, Adolph RJ, DiMarco JP, Dreifus LS, Dunn MI, Fisch C, et al. Guidelines for electrocardiography. A report of the American College of Cardiology/American Heart Association task force on assessment of diagnostic and therapeutic cardiovascular procedures (committee on electrocardiography). Circulation. 1992;85(3):1221–8. 10.1161/01.cir.85.3.1221 PMID: 1537123. 10.1161/01.cir.85.3.1221 1537123

[11] Chan YH (2003). Biostatistics 102: quantitative data - Parametric & non-parametric Tests. Singap Med J.

[12] Chan YH (2003). Biostatistics 103: qualitative data –tests of Independence. Singap Med J.

[13] Eckner D, Pollari F, Santarpino G, et al. Comparison between Surgical Access and Percutaneous Closure Device in 787 Patients Undergoing Transcatheter Aortic Valve Replacement. J Clin Med. 2021;10(7):1344. Published 2021 Mar 24. 10.3390/jcm10071344. 10.3390/jcm10071344 PMC8037566 33805069

[14] Lee CH, Huang JK, Yang TF (2016). Use of a Perclose ProGlide device for percutaneous endovascular aortic aneurysm repair in a general hospital experience. Discov Med.

[15] Hu G, Chen B, Fu W, et al. Predictors and treatments of Proglide-related complications in percutaneous endovascular aortic repair. PLoS One. 2015;10(4):e0123739. Published 2015 Apr 22. 10.1371/journal.pone.0123739. 10.1371/journal.pone.0123739 PMC4406497 25901610

[16] Ichihashi T, Ito T, Kinoshita Y, Suzuki T, Ohte N (2016). Safety and utility of total percutaneous endovascular aortic repair with a single Perclose ProGlide closure device. J Vasc Surg.

[17] Drafts BC, Choi CH, Sangal K (2018). Comparison of outcomes with surgical cut-down versus percutaneous transfemoral transcatheter aortic valve replacement: TAVR transfemoral access comparisons between surgical cut-down and percutaneous approach. Catheter Cardiovasc Interv.

[18] Al-Khatib WK, Zayed MA, Harris EJ, Dalman RL, Lee JT (2012). Selective use of percutaneous endovascular aneurysm repair in women leads to fewer groin complications. Ann Vasc Surg.

[19] Nelson PR, Kracjer Z, Kansal N (2014). A multicenter, randomized, controlled trial of totally percutaneous access versus open femoral exposure for endovascular aortic aneurysm repair (the PEVAR trial). J Vasc Surg.

[20] Sandoval Y, Burke MN, Lobo AS (2017). Contemporary Arterial Access in the Cardiac Catheterization Laboratory. JACC Cardiovasc Interv.

